# Ecological analysis of Kashin-Beck osteoarthropathy risk factors in Tibet’s Qamdo City, China

**DOI:** 10.1038/s41598-019-39792-0

**Published:** 2019-02-21

**Authors:** Xinjie Zha, Xing Gao

**Affiliations:** 1 State Key Laboratory of Resources and Environmental Information System, Institute of Geographic Sciences and Natural Resources Research, Chinese Academy of Sciences, Beijing, 100101 China; 2 University of Chinese Academy of Sciences, Beijing, 100049 China

## Abstract

We studied Tibet’s Qamdo City, which currently hosts the most serious prevalence of Kashin-Beck osteoarthropathy (KB) in China. This study utilizes the geographical detector (GeoDetector) algorithm to measure the individual and interactive influences of risk factors on KB and to quantify the highest potential risk subzones of each principal factor. With a comprehensive consideration of 13 possible related factors, namely, the tectonic division, stratum, moisture index, gross domestic product, mean annual precipitation, soil type, groundwater type, elevation, mean annual temperature, vegetation type, geomorphic type, slope degree and slope aspect, our results indicate that the main exposure factors for KB in Qamdo City are geological factors (tectonic division and stratum), wetting factors (moisture index and mean annual precipitation), and an economic factor (gross domestic product). In contrast, other factors have little effect on the prevalence of KB in Qamdo City. All 13 factors either nonlinearly or bivariately enhance each other, and the interactions between these factors can increase the prevalence of KB. Consequently, it can be inferred that KB in Qamdo City is caused primarily by a set of multiple and interrelated disease risk factors.

## Introduction

Kashin-Beck osteoarthropathy (KB), which mainly afflicts certain areas ranging from southeastern Siberia to northern China, North Korea, central China and Tibet^1–6^, is an endemic disabling osteoarticular disease involving growth cartilage that results in severe alteration of the joints. The most serious and active area of KB in China is currently Tibet, where the most serious disease conditions are observed in Qamdo City^5–10^.

Three major environmental etiological hypotheses, namely, biogeochemical imbalance (endemic selenium (Se) deficiency)^11–16^, food fungi toxin poisoning (serious cereal contamination by mycotoxin-producing fungi)^17–20^, and water organic compound poisoning (high humic acid levels in drinking water)^21–24^, have been proposed as the causes of KB; however, the specific etiology of the disease is still unknown. Moreover, some difficult questions continue to arise with these hypotheses. For instance, the biogeochemical hypothesis cannot explain why people not suffer from KB in some areas of low Se, while KB has occurred in some locations where the level of Se is not very low^25–27^; furthermore, a random clinical trial consisting of iodine and Se supplementation with a one-year duration neither clinically nor radiologically improved the evolution of KB in children^6,28^. Meanwhile, neither food fungi toxin poisoning nor water organic compound poisoning can explain the focal distribution pattern of KB endemic areas in close range^29^.

Zhang, *et al*.^30^ analyzed the environmental levels of Se in Rangtang County and Aba County, both of which are part of the Qinghai-Tibet Plateau, where soil Se deficiency plays a critical role in the local prevalence of KB, and they generally found that the prevalence of KB is higher for lower levels of environmental Se. Wang, *et al*.^31^ investigated the distribution and translocation of Se from soil to highland barley in both non-KB and KB endemic areas, and they showed that the Se concentration in highland barley was too low to meet the minimum human daily intake requirements. Zhao, *et al*.^32^ believed that the etiology of KB in the Qinghai-Tibet Plateau was unclear and that Se deficiency was not the real cause; alternatively, they postulated that Se deficiency was an important environmental risk factor. Moreno-Reyes, *et al*.^33^ evaluated the iodine and Se status in 575 Tibetan children with KB, and they found that iodine deficiency was a risk factor for KB in areas with a severe Se deficiency. Yang, *et al*.^34,35^ discussed how land use patterns and soil types affect the distribution of KB in Qamdo City and reported that areas most severely afflicted with the disease are located in agricultural and semi-agricultural counties with forest brown and cinnamon forest soil types. Chasseur, *et al*.^36,37^ reported that grains contaminated by fungi during their drying and storage in humid environments were the cause for the proliferation of KB throughout Tibet. Yang, *et al*.^38^ also studied the temperature, geology and altitude in Tibet as the influencing factors on KB by overlaying different spatial distribution maps and noted that Qamdo City, which is the most seriously diseased area, is located in an alpine temperate semi-humid region. Although researchers have increasingly tended toward concluding that KB is caused by a set of multiple and interrelated environmental factors^39–42^, the above mentioned studies failed to conduct a quantitative analysis on the combined effects of multiple important factors under a unified framework. In general, some related factors, including geological, geographical, ecological, climatic and economic factors, have the potential to become KB determinants. Therefore, by exploring the relationships between these factors and KB, we may be able to understand the main exposure factors for KB.

Geological, geographical, ecological, climatic and economic phenomena are characterized by two major features^43^: spatial autocorrelation (two close geographical sites are more similar or more dissimilar than two distant sites)^44^ and spatial stratified heterogeneity (the within-strata variance is less than the between-strata variance)^45^. Based on the existence of spatial stratified heterogeneity, the GeoDetector algorithm designed by Wang, *et al*.^46,47^ can utilize the spatial variance to quantify the relative importance of single factors and their implicit interactions with response variables. Consequently, the GeoDetector method can be employed to measure the spatial consistency and statistical significance between KB prevalence and suspicious influencing factors^48^.

**Figure 1:**
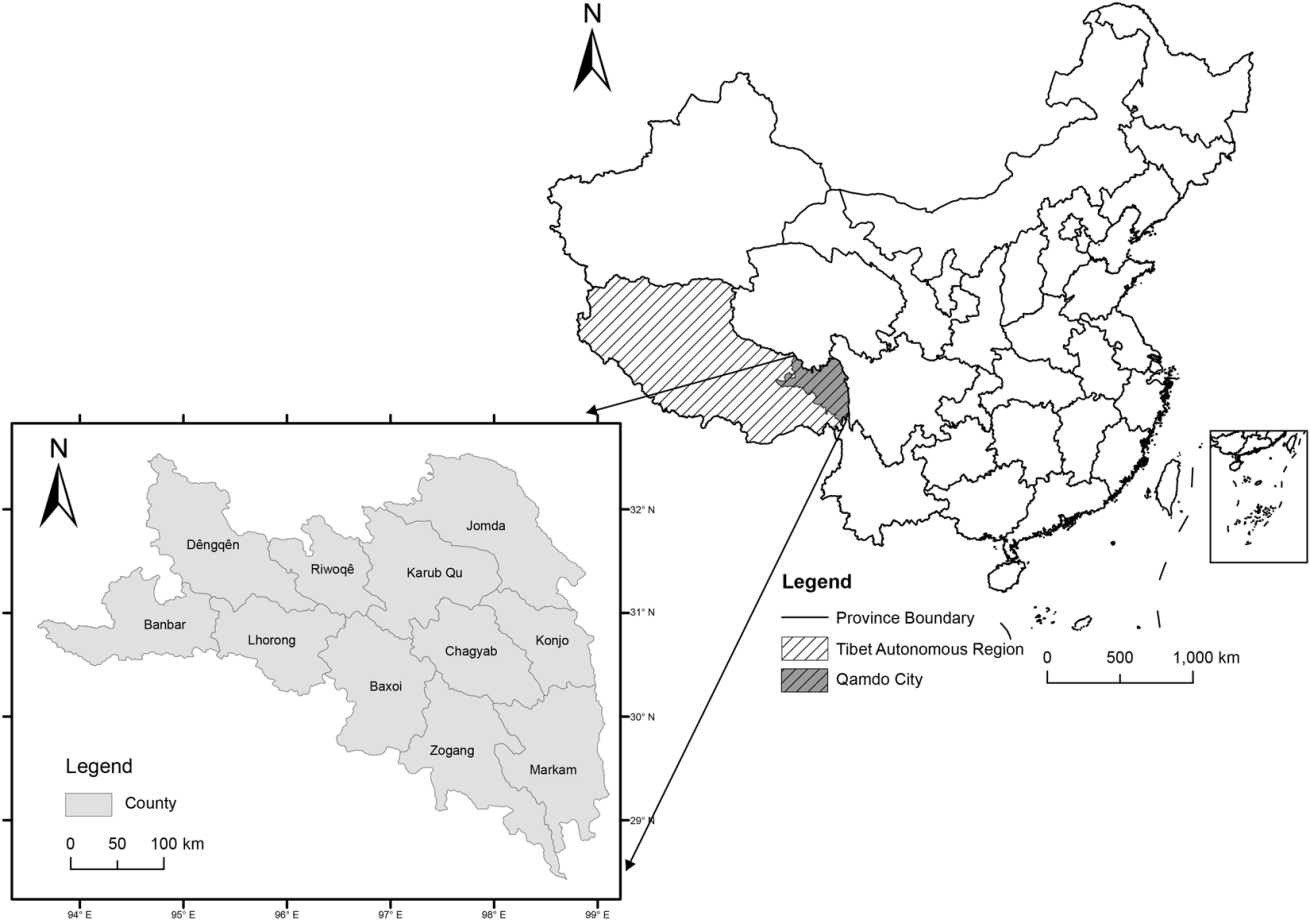
Location map for the study area showing the 11 counties in Tibet’s Qamdo City. The map was generated by ArcGIS 10.2 (http://www.esri.com/).

Many scholars have studied some possible causes of KB, but a quantitative analysis on the interactive effects of multiple important factors is lacking. Therefore, based on the concept of spatial stratified heterogeneity and by using the GeoDetector method, the objectives of this study are to answer the following questions: (1) What are the main exposure factors for KB in Qamdo City? (2) Do these factors operate independently, or are they interconnected? (3) What are the relatively important subzones of each risk factor? Tibet’s Qamdo City was taken as the case study in this research. Qamdo City (93°E to 99°E and 28°N to 32°N) is located in eastern Tibet within the Hengduan Mountain region and Three-River Valley (composed of the Jinsha River, Lancang River, and Nu River) with an area of 1.07 × 10^5^ km^2^ (Fig. 1); the mean elevation is approximately 4453.66 m. The annual mean temperature is 6.45 °C, and the annual mean precipitation is 585.35 mm.

## Results

### Relative influences of risk factors on KB

**Figure 2:**
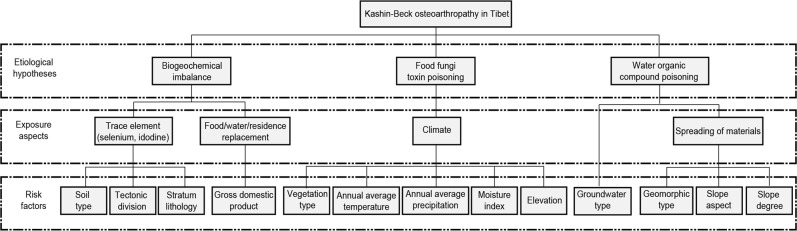
The relationships among the risk factors, exposure aspects and etiological hypotheses.

The factor detector quantifies the potential risk factors of KB on the basis of their q-statistic values. For this purpose, we selected the tectonic division (TEC), stratum (STR), moisture index (MI), gross domestic product (GDP), mean annual precipitation (PRE), soil type (SOI), groundwater type (GRO), elevation (ELE), mean annual temperature (TEM), vegetation type (VEG), geomorphic type (GEO), slope degree (SD) and slope aspect (SA) as the 13 potential disease risk factors to study their individual influences on the prevalence of KB (Fig. 2).

**Figure 3:**
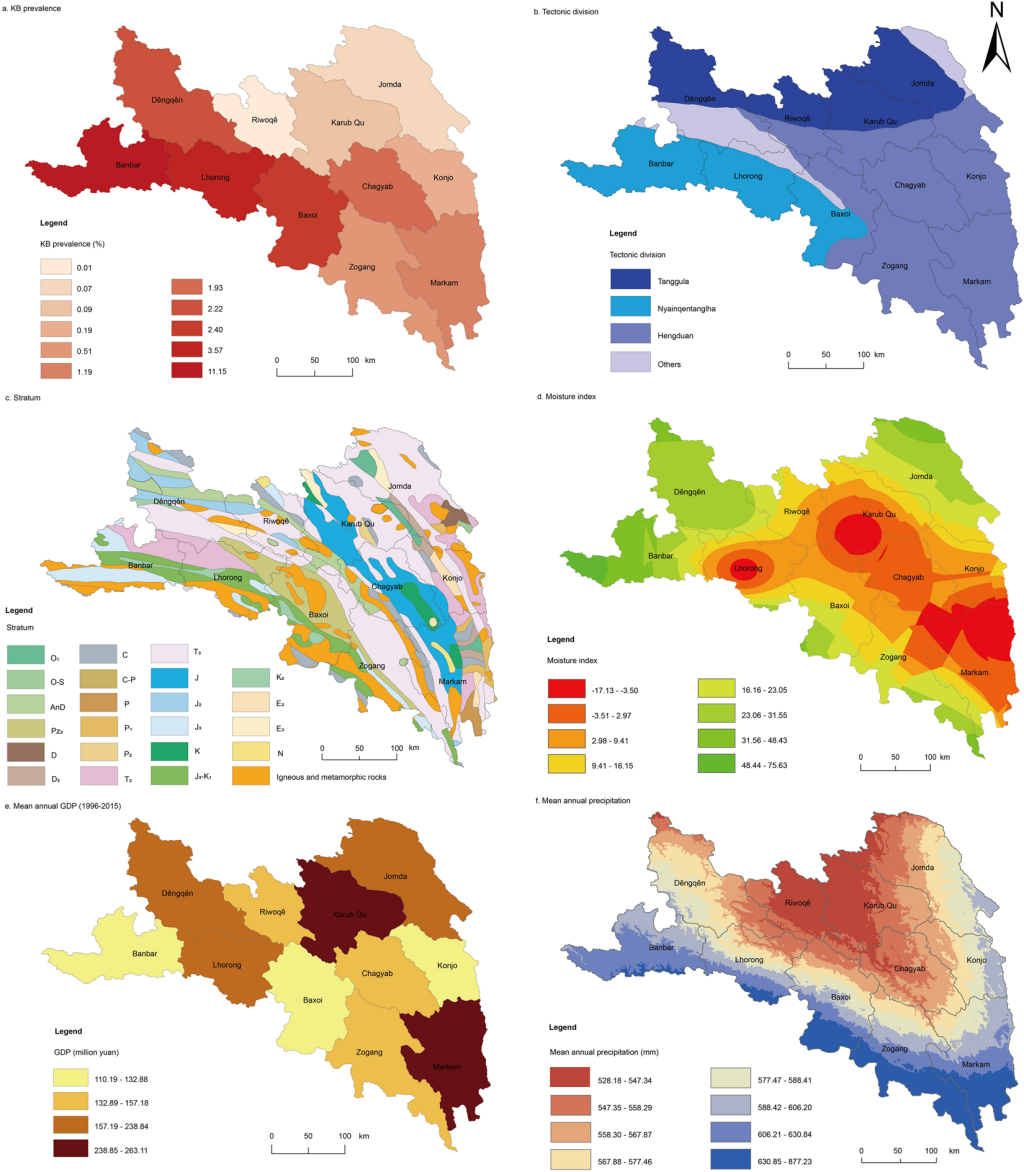
Spatial distributions of the prevalence rate and five main potential risk factors of KB in Qamdo City. The maps were generated by ArcGIS 10.2 (http://www.esri.com/). (**a**) KB prevalence rates of 11 counties; (**b**) Tectonic division of Qamdo City containing 4 types of vector data; (**c**) Stratum map of Qamdo City containing 23 types of vector data; (**d**) Moisture index map of Qamdo City classified into 8 types through the natural break method to reclassify the raster data; (**e**) Data of the gross domestic product divided into quartiles; (**f**) Mean annual precipitation calculated by the ANUSPLINE software and classified into 8 quantiles to reclassify the raster data.

The q-statistic values of the factors are sorted as follows: TEC (0.560) > STR (0.467) > MI (0.334) > GDP (0.314) > PRE (0.294) > SOI (0.117) > GRO (0.088) > ELE (0.081) > TEM (0.051) > VEG (0.043) > GEO (0.042) > SD (0.012) > SA (0.003). Among these factors, the q-statistic values of TEC and STR are larger than those of the other factors. Generally, if the q-statistic value of a factor is larger than 0.2, it can explain the spatial pattern well, and thus, this factor can be regarded as an important potential risk factor^49^. Therefore, TEC (0.560), STR (0.467), MI (0.334), GDP (0.314) and PRE (0.294) are the most important potential risk factors, and they have larger impacts on the prevalence rate of KB in this study area (Fig. 3). Furthermore, the value of SOI (0.117) is between 0.1 and 0.2, and values of GRO (0.088), ELE (0.081), TEM (0.051), VEG (0.043), GEO (0.042), SD (0.012) and SA (0.003) are all less than 0.1, meaning that they have little impact on the prevalence of KB.

### Statistically significant differences among the risk factors of KB

The ecological detector identifies the differences in the impacts between any two impact factors of KB. The results reveal that the differences between any two risk factors are not statistically significant at a confidence level of 95%, meaning that no single factor has a significantly larger impact on the prevalence of KB^43^. Based on a comprehensive consideration of the results from the factor detector and the ecological detector, we concluded that TEC, STR, MI, GDP, and PRE are likely to constitute the dominant risk factors and have the largest impact on the prevalence of KB; in contrast, the influences of the remaining factors are relatively weak.

### Interactive influences of individual risk factors on KB

The interaction detector was used to reveal the interaction relationship between two factors regardless of whether they work independently. The detection results of every pair of factors with interactive q-statistic values > 0.6 are tabulated in Table 1 (all interactive pairs are tabulated in Supplementary Table S1).

**Table 1:** Interactive q-statistic values (>0.6) between pairs of risk factors.

C = A ∩ B	A	B	D = A + B	Result	Interaction
TEC ∩ MI = 0.777	0.560	0.334	0.894	C < D; C > Max(A,B)	↑
MI ∩ GDP = 0.760	0.334	0.314	0.648	C > D; C > Max(A,B)	↑↑
TEC ∩ STR = 0.731	0.560	0.467	1.027	C < D; C > Max(A,B)	↑
STR ∩ GDP = 0.710	0.467	0.314	0.781	C < D; C > Max(A,B)	↑
MI ∩ PRE = 0.689	0.334	0.294	0.628	C > D; C > Max(A,B)	↑↑
STR ∩ MI = 0.682	0.467	0.334	0.801	C < D; C > Max(A,B)	↑
STR ∩ PRE = 0.679	0.467	0.294	0.761	C < D; C > Max(A,B)	↑
TEC ∩ PRE = 0.674	0.560	0.294	0.854	C < D; C > Max(A,B)	↑
TEC ∩ GDP = 0.673	0.560	0.314	0.874	C < D; C > Max(A,B)	↑
TEC ∩ TEM = 0.640	0.560	0.051	0.611	C > D; C > Max(A,B)	↑↑
GDP ∩ PRE = 0.628	0.314	0.294	0.608	C > D; C > Max(A,B)	↑↑
TEC ∩ SOI = 0.614	0.560	0.117	0.677	C < D; C > Max(A,B)	↑

^“↑”^Denotes the bivariate enhancement of A and B; ^“↑↑”^denotes the nonlinear enhancement of A and B. The risk factors include the following: TEC, STR, MI, GDP, PRE, SOI and TEM.

We found that the interaction between any two risk factors enhanced their impact on the prevalence of KB with q-statistic values that are higher than those of the individual factors; this reveals that the two factors enhance each other and that this occurs even for those factors with the lowest q-statistic values. Among all possible pairs of the 13 factors, the q-statistic values of 57 interactive pairs are larger than 0.2. The q-statistic values of the interactive pairs TEC and MI, MI and GDP, TEC and STR, and STR and GDP are larger than 0.7, and the largest interactive value is 0.777 (TEC ∩ MI = 0.777). There are two enhancement types: nonlinear enhancement and bivariate enhancement (Table 1), which indicate that the two factors either nonlinearly enhance or bivariately enhance each other, respectively. For instance, the interactive q-statistic value between TEC and MI is larger than the maximum q-statistic of TEC and MI, but both factors are smaller than the summed q-statistic value of TEC and MI, which means that TEC and MI bivariately enhance each other (Max (TEC, MI) = 0.560 < TEC ∩ MI = 0.777 < TEC + MI = 0.894). In the case of nonlinear enhancement, for example, the interactive q-statistic between MI and PRE is larger than the summed q-statistic of the two factors (MI ∩ PRE = 0.689 > 0.628 = MI + PRE); therefore, the interaction between MI and PRE has a larger impact on KB.

### Regional analysis of the potential impact ranges (types) of risk factors on KB

The risk detector reveals the average prevalence of KB in the subzone of each risk factor and indicates the main impact type or range. The main impact ranges or types of the five most important potential risk factors are Nyainqêntanglha (TEC), Upper Jurassic (STR), 48.43–75.63 (MI), 110.19–132.88 million yuan (GDP), and 606.20–630.84 mm (PRE), as tabulated in Table 2. These findings indicate that these ranges or types of these five risk factors probably lead to a higher prevalence of KB than those of the other risk factors.

**Table 2:** Main impact ranges/types of factors on KB in the study area.

Factor	Impact type/range	KB average prevalence (%)
Tectonic division	Nyainqêntanglha	6.58
Stratum	Upper Jurassic J3	7.70
Moisture index	48.43–75.63	11.15
GDP (million yuan)	110.19–132.88	4.91
Mean annual precipitation (mm)	606.20–630.84	5.80

## Discussion and Conclusions

Although several etiological hypotheses have been proposed worldwide since KB was first documented and reported in 1849, the pathogeny of the disease remains uncertain. In this study, the GeoDetector method was used to provide an objective and quantitative analytical framework with which to investigate the independent and interactive impacts of potential factors on the prevalence of KB. Meanwhile, the GeoDetector algorithm also discovered the main risk types or ranges of the KB exposure factors, which are important for studying the pathogenesis of KB.

This study indicates that the variation in KB was predominantly and significantly impacted (either independently or jointly) by the tectonic division, stratum, moisture index, GDP, and mean annual precipitation. However, the soil type showed a relatively low individual influence, and the groundwater type, mean annual temperature, vegetation type, geomorphology and topographic factors (including the elevation, slope degree, and slope aspect) had the lowest individual q-statistic values. In addition, we also confirmed that the interactions between pairs of factors played more important roles than their individual effects on the variation in KB. Although the individual effects of the groundwater type, mean annual temperature and vegetation type were weak, these factors had greater influences on the prevalence of KB when interacting with the tectonic division, stratum, and moisture index.

From the GeoDetector results, the tectonic division (q-statistic = 0.560) represents the leading exposure factor. There are three major tectonic divisions in Qamdo City, namely, Hengduan, Nyainqêntanglha and Tanggula, and the highest average prevalence rate of KB was located in the tectonic division of Nyainqêntanglha. The tectonic division essentially reflects the regional geological structure. Yang, *et al*.^38^ reported that KB in the Qinghai-Tibet Plateau was mainly distributed throughout the northern section of the Hengduan Mountains and between the Himalayas and the Gangdise-Nyainqêntanglha Mountains. Therefore, the distribution of KB is mainly affected and controlled by the regional geological structure.

The stratum appeared to be the second-most important exposure factor for KB with an individual q-statistic value of 0.467. Se deficiency within a stratum can lead to a lack of selenium in weathered soils, and leading to a low Se nutritional status of the local populace through the food chain^12,19,50^. We found that the average prevalence rates of KB in different strata are different; the top three are the Upper Jurassic (J_3_, 145 Ma–163.5 Ma), Lower Cretaceous (K_1_, 100.5 Ma–145 Ma) and Upper Cretaceous (K_2_, 66 Ma–100.5 Ma). Large, *et al*.^51^ calculated the average concentrations of Se in pyrite samples with 84 ages, and the concentrations of Se in J_3_ (145 Ma = 4.63 mg/kg), K_1_ (142 Ma = 5.92 mg/kg, 131 Ma = 3.24 mg/kg) and K_2_ (92 Ma = 3.54 mg/kg, 83 Ma = 4.13 mg/kg) were significantly lower than the average Se concentration (138.85 mg/kg) of the 2119 samples from all ages (0.1 Ma–560 Ma). In addition, the tectonic division and stratum factors, which can be classified as geological factors, are enhanced and have greater effects on each other when interacting (TEC ∩ STR = 0.731). These results further confirm that geological factors could be the most important exposure factors leading to the occurrence of KB.

The moisture index and mean annual precipitation had relatively high q-statistic values of 0.334 and 0.294, respectively. There was also a significant correlation between the moisture index and precipitation (r = 0.56); thus, these two factors could be classified as wetting factors. The interactive effects of these two factors also significantly enhanced the prevalence of KB. Meanwhile, the average prevalence rate of KB was different under the different values of the moisture index and mean annual precipitation, and the highest prevalence of classification was 48.43–75.63 (the largest classification interval of moisture index, a total of 8 intervals) and 606.20 mm–630.84 mm (the second-largest classification interval of the mean annual precipitation, a total of 8 intervals), respectively. Thus, these two factors could provide a appropriate explanation for the food fungi toxin poisoning hypothesis, in which food is contaminated by fungi under wet conditions during drying and storage processes^37^.

The q-statistic of the GDP was 0.314, indicating that the GDP factor had some influence on the prevalence of KB. Relatively poor families originated mainly from agricultural areas or semi-agricultural and semi-pastoral areas, had a single diet and relied on their own planted highland barley and raised livestock. Some studies also confirmed that the prevalence rate of KB reduced after a period of time when some Tibetan villagers afflicted with KB changed their water and food sources or moved to a new environment^52^. However, due to the local geological and geographical background, people often lack the monetary resources to change their water and food sources or move to a new environment, and thus, they continue to suffer from KB.

By using the GeoDetector method to study multiple and interrelated potential exposure factors for KB, we can draw some conclusions. (1) The main exposure factors of KB in Qamdo City are geological factors (TEC and STR) and wetting factors (MI and PRE) in addition to an economic factor (GDP); the main reasons for these factors could be low-Se strata controlled by regional geological structures and high-humidity environments controlled by precipitation and evaporation in addition to a lack of money for changing the sources of water and food or moving to a new environment. (2) Some factors, including the groundwater type, mean annual temperature, vegetation type, geomorphic type, elevation, slope degree, and slope aspect, have little effect on the prevalence of KB in Qamdo City. (3) All 13 factors either nonlinearly or bivariately enhance each other, and the interactions between these factors can enhance the prevalence rate of KB. (4) It can be inferred that the prevalence of KB in Qamdo City is caused by multiple interrelated factors. To some extent, our work may be helpful for understanding the various impact factors on KB in Qamdo City. However, some issues must yet be resolved. In this study, only 13 relevant factors were considered for the prevalence of KB in Qamdo City; these factors seem to be insufficient for capturing the complexity of the pathogenesis of KB. Additional exposure factors should be studied in the future, which will also contribute to ongoing research on the pathogenesis of KB.

## Methods and Materials

### Research methods

The geographical detector, which is composed of a factor detector, an ecological detector, a risk detector, and an interaction detector, represents a new spatial analysis method for the assessment of health risks based on spatial stratified heterogeneity^45,46,53,54^. The more identical the spatial distribution of any pair of variables, the higher the association is between the two variables. Furthermore, the spatial distribution of a factor will show greater consistency with changes in geographical phenomena.

In our study, we assumed that the prevalence of KB would show a similar spatial distribution to certain factors if those factors mainly contribute to the prevalence of the disease. All impact factors are quantified by the following q-statistic values (*P*_*D,H*_), Eq. (1) as follows:

1 $$q={P}_{D,H}=1-\frac{1}{n{\sigma }_{H}^{2}}\sum _{i=1}^{m}({n}_{D,i}\cdot {\sigma }_{{H}_{D,i}}^{2})$$

where *H* represents the prevalence rate of KB; *D* = {*D*_*i*_} represents the disease risk factors; *P*_*D,H*_ represents the power of the determinant *D* on *H*; *n* and ${\sigma }_{H}^{2}$ represent the number of total samples and the variance of *H* in the study area, respectively; and the dispersion variances of *H* over the sub-regions of the attributes *D*_*i*_ are denoted as ${\sigma }_{{H}_{D,i}}^{2}$. The range of the q-statistic value (*P*_*D,H*_) is [0, 1]^46,55^; if the prevalence of KB is completely associated with one risk factor, then *q* = 1, whereas *q* = 0 implies the lack of an association between the prevalence of KB and a risk factor, i.e., a completely random spatial occurrence of KB. A higher value indicates a higher spatial association, and the q-statistic represents how much the prevalence of KB is determined by one risk factor. More details on GeoDetector can be found in the original paper^45,46^, and a free version of GeoDetector Software in R can be downloaded from http://www.geodetector.org.

### Data sources

All KB cases were verified by doctors in the hospital and county health bureau. Records of KB cases in Qamdo City were obtained from the 2015 local annual report of the Tibet Autonomous Region Centers for Disease Control and Prevention, Health and Family Planning Commission of Tibet (Table 3). The local health and family planning department declined to provide identifiers to link substantiated KB cases, and thus, we were unable to accurately match case data to spatial points; hence, we aggregated the KB cases by county rather than at the individual level.

**Table 3:** Number of KB observations and cases and the KB prevalence of each county in Qamdo.

County name	Observation population (ten thousand)	KB-afflicted administrative villages	KB cases	KB prevalence (%)
Karub Qu	12.49	7	114	0.09
Konjo	4.30	2	81	0.19
Lhorong	4.86	25	1,734	3.57
Jomda	8.40	20	56	0.07
Dêngqên	7.60	8	1,686	2.22
Riwoqê	5.00	1	5	0.01
Zogang	4.70	33	242	0.51
Chagyab	5.70	12	1,100	1.93
Markam	9.80	14	1,163	1.19
Banbar	3.60	69	4,013	11.15
Baxoi	4.20	37	1,010	2.40
Total	70.65	228	11,204	1.59

The mean annual precipitation (PRE) data recorded at 55 meteorological stations from 1982 to 2015 in the Tibetan Plateau (Fig. 3(f)) were interpolated with the ANUSPLINE software, which was programmed by Hutchinson^56^, and then the data for Qamdo City were extracted with ArcGIS 10.2. The above data were obtained from the China Meteorological Data Service Center (http://data.cma.cn).

Topographic factors, including the elevation (ELE) (Fig. S3), slope degree (SD) (Fig. S7), and slope aspect (SA) (Fig. S8) were extracted from the 90 m Shuttle Radar Topography Mission (SRTM) digital elevation model (DEM) using ArcGIS 10.2. Vegetation type (VEG) data were available from the vegetation type map of China (scale 1:1000000) (Fig. S5). Soil type (SOI) data were digitized from the soil type map of China (scale 1:1000000) (Fig. S1). Geomorphic type (GEO) data were derived from geomorphic map of China (scale 1:1000000) (Fig. S6). Mean annual temperature (TEM) data were corrected by a DEM (scale 1:1000000) (Fig. S4). Moisture index (MI) data were interpolated using the inverse distance weighting method (scale 1:1000000) (Fig. 3). All of the above data were obtained from the Data Centre for Resources and Environmental Sciences, Chinese Academy of Sciences (RESDC) (http://www.resdc.cn).

Groundwater (GRO) data (scale 1:4000000) were obtained by the Institute of Hydrogeology and Environmental Geology, Chinese Academy of Geological Sciences (Fig. S2), Tectonic division (TEC) data (scale 1:4000000) were drawn by the Lanzhou Institute of Glaciology and Cryopedology, Chinese Academy of Sciences (Fig. 3(b)). Stratum data (scale 1:5000000) were drawn by the Ministry of Geology and Mineral Resources of the People’s Republic of China (Fig. 3(c)). All of the above data were obtained from the Digital Library of National Geological Archives of China (http://www.ngac.org.cn).

Gross domestic product (GDP) data were derived from the Tibet Statistical Yearbooks (1996–2015), and the mean annual GDP was calculated for each county in Qamdo City from 1996 to 2015 (Fig. 3(e)).

KB prevalence data were adjusted by the empirical Bayes smoothing method in GeoDa (https://spatial.uchicago.edu/software), and thus, the problem of a small sample size was alleviated. All data were extracted in a fishnet grid (2 km × 2 km) with the intersect analysis tool in ArcMap and then input into GeoDetector Software in R.

## Supplementary information

Supplementary Information
