# Supplementary material for: Ecological analysis of Kashin-Beck osteoarthropathy risk factors in Tibet’s Qamdo City, China
Source: Sci Rep. 2019 Feb 21;9:2471. doi: 10.1038/s41598-019-39792-0 (PMC6385338; doi:10.1038/s41598-019-39792-0)
Supplement: Supplementary file 1 — Supplementary Information [file 41598_2019_39792_MOESM1_ESM.pdf]

Ecological analysis of Kashin-Beck osteoarthropathy risk factors in Tibet's Qamdo Prefecture, China

Xinjie Zha[1, 2], Xing Gao[1,*]

[1]State Key Laboratory of Resources and Environmental Information System, Institute of Geographic Sciences and Natural Resources Research, Chinese Academy of Sciences, Beijing 100101, China

[2]University of Chinese Academy of Sciences, Beijing 100049, China

*Corresponding. gxing@igsnrr.ac.cn

**C=A∩B**	**A**	**B**	**D=A+B**	**Result**	**Interaction**
TEC∩MI=0.777	0.560	0.334	0.894	C<D; C>Max(A,B)	↑
MI∩GDP=0.760	0.334	0.314	0.648	C>D; C>Max(A,B)	↑↑
TEC∩STR=0.731	0.560	0.467	1.027	C<D; C>Max(A,B)	↑
STR∩GDP=0.710	0.467	0.314	0.781	C<D; C>Max(A,B)	↑
MI∩PRE=0.689	0.334	0.294	0.628	C>D; C>Max(A,B)	↑↑
STR∩MI=0.682	0.467	0.334	0.801	C<D; C>Max(A,B)	↑
STR∩PRE=0.679	0.467	0.294	0.761	C<D; C>Max(A,B)	↑
TEC∩PRE=0.674	0.560	0.294	0.854	C<D; C>Max(A,B)	↑
TEC∩GDP=0.673	0.560	0.314	0.874	C<D; C>Max(A,B)	↑
TEC∩TEM=0.640	0.560	0.051	0.611	C>D; C>Max(A,B)	↑↑
GDP ∩PRE=0.628	0.314	0.294	0.608	C>D; C>Max(A,B)	↑↑
TEC∩SOI=0.614	0.560	0.117	0.677	C<D; C>Max(A,B)	↑
ELE∩TEC=0.597	0.560	0.081	0.641	C<D; C>Max(A,B)	↑
VEG∩TEC=0.595	0.560	0.043	0.603	C<D; C>Max(A,B)	↑
GEO∩TEC=0.592	0.560	0.042	0.602	C<D; C>Max(A,B)	↑
GRO∩TEC=0.573	0.560	0.088	0.648	C<D; C>Max(A,B)	↑
GEO∩STR=0.573	0.467	0.042	0.509	C>D; C>Max(A,B)	↑↑
SD∩TEC0.563	0.560	0.012	0.572	C<D; C>Max(A,B)	↑
SA∩TEC=0.562	0.560	0.003	0.563	C<D; C>Max(A,B)	↑
GRO∩STR=0.556	0.467	0.088	0.555	C>D; C>Max(A,B)	↑↑
TEM∩STR=0.55	0.467	0.051	0.518	C>D; C>Max(A,B)	↑↑
SOI∩STR=0.545	0.467	0.117	0.584	C<D; C>Max(A,B)	↑
ELE∩STR=0.534	0.467	0.081	0.548	C<D; C>Max(A,B)	↑
GRO∩GDP=0.518	0.314	0.088	0.402	C>D; C>Max(A,B)	↑↑
VEG∩STR=0.514	0.467	0.043	0.510	C>D; C>Max(A,B)	↑↑
SD∩STR=0.482	0.467	0.012	0.479	C>D; C>Max(A,B)	↑↑
SA∩STR=0.477	0.467	0.003	0.470	C>D; C>Max(A,B)	↑↑

GRO∩PRE=0.456	0.294	0.088	0.382	C>D; C>Max(A,B)	↑↑
TEM∩PRE=0.428	0.294	0.051	0.345	C>D; C>Max(A,B)	↑↑
SOI∩PRE=0.419	0.294	0.117	0.411	C>D; C>Max(A,B)	↑↑
GEO∩PRE=0.416	0.294	0.042	0.336	C>D; C>Max(A,B)	↑↑
GEO∩GDP=0.412	0.314	0.042	0.356	C>D; C>Max(A,B)	↑↑
SOI∩MI=0.405	0.334	0.117	0.451	C<D; C>Max(A,B)	↑
SOI∩GDP=0.391	0.314	0.117	0.431	C<D; C>Max(A,B)	↑
VEG∩GDP=0.391	0.314	0.043	0.357	C>D; C>Max(A,B)	↑↑
ELE∩PRE=0.39	0.294	0.081	0.375	C>D; C>Max(A,B)	↑↑
GRO∩MI=0.385	0.334	0.088	0.422	C<D; C>Max(A,B)	↑
TEM∩GDP=0.385	0.314	0.051	0.365	C>D; C>Max(A,B)	↑↑
VEG∩MI=0.384	0.334	0.043	0.377	C>D; C>Max(A,B)	↑↑
GEO∩MI=0.382	0.334	0.042	0.376	C>D; C>Max(A,B)	↑↑
ELE∩MI=0.374	0.334	0.081	0.415	C<D; C>Max(A,B)	↑
TEM∩MI=0.371	0.334	0.051	0.385	C<D; C>Max(A,B)	↑
ELE∩GDP=0.369	0.314	0.081	0.395	C<D; C>Max(A,B)	↑
SD∩MI=0.347	0.334	0.012	0.346	C>D; C>Max(A,B)	↑↑
SD∩GDP=0.343	0.314	0.012	0.326	C>D; C>Max(A,B)	↑↑
SA∩MI=0.342	0.334	0.003	0.337	C>D; C>Max(A,B)	↑↑
VEG∩PRE=0.341	0.294	0.043	0.337	C>D; C>Max(A,B)	↑↑
SA∩GDP=0.319	0.314	0.003	0.317	C>D; C>Max(A,B)	↑↑
SA∩PRE=0.304	0.294	0.003	0.297	C>D; C>Max(A,B)	↑↑
SD∩PRE=0.302	0.294	0.012	0.306	C<D; C>Max(A,B)	↑
GEO∩SOI=0.19	0.117	0.042	0.159	C>D; C>Max(A,B)	↑↑
VEG∩SOI=0.168	0.117	0.043	0.160	C>D; C>Max(A,B)	↑↑
GRO∩SOI=0.167	0.117	0.088	0.205	C<D; C>Max(A,B)	↑
ELE∩SOI=0.155	0.117	0.081	0.198	C<D; C>Max(A,B)	↑
GEO∩GRO=0.155	0.088	0.042	0.130	C>D; C>Max(A,B)	↑↑
GEO∩ELE=0.154	0.081	0.042	0.123	C>D; C>Max(A,B)	↑↑
TEM∩SOI=0.151	0.117	0.051	0.168	C<D; C>Max(A,B)	↑
VEG∩GRO=0.146	0.088	0.043	0.131	C>D; C>Max(A,B)	↑↑
SD∩SOI=0.144	0.117	0.012	0.129	C>D; C>Max(A,B)	↑↑
TEM∩ELE=0.133	0.081	0.051	0.132	C>D; C>Max(A,B)	↑↑
ELE∩GRO=0.131	0.088	0.081	0.169	C<D; C>Max(A,B)	↑
VEG∩ELE=0.126	0.081	0.043	0.124	C>D; C>Max(A,B)	↑↑
SA∩SOI=0.122	0.117	0.003	0.120	C>D; C>Max(A,B)	↑↑
SD∩ELE=0.119	0.081	0.012	0.093	C>D; C>Max(A,B)	↑↑
TEM∩GRO=0.117	0.088	0.051	0.139	C<D; C>Max(A,B)	↑
GEO∩TEM=0.114	0.051	0.042	0.093	C>D; C>Max(A,B)	↑↑
VEG∩TEM=0.102	0.051	0.043	0.094	C>D; C>Max(A,B)	↑↑
SD∩GRO=0.101	0.088	0.012	0.100	C>D; C>Max(A,B)	↑↑
GEO∩VEG=0.098	0.043	0.042	0.085	C>D; C>Max(A,B)	↑↑

SA∩GRO=0.09	0.088	0.003	0.091	C<D; C>Max(A,B)	↑
SD∩TEM=0.086	0.051	0.012	0.063	C>D; C>Max(A,B)	↑↑
SA∩ELE=0.085	0.081	0.003	0.084	C>D; C>Max(A,B)	↑↑
SD∩GEO=0.074	0.042	0.012	0.054	C>D; C>Max(A,B)	↑↑
SA∩TEM=0.059	0.051	0.003	0.054	C>D; C>Max(A,B)	↑↑
SD∩VEG=0.053	0.043	0.012	0.055	C<D; C>Max(A,B)	↑
SA∩GEO=0.05	0.042	0.003	0.045	C>D; C>Max(A,B)	↑↑
SA∩VEG=0.048	0.043	0.003	0.046	C>D; C>Max(A,B)	↑↑
SA∩SD=0.018	0.012	0.003	0.015	C>D; C>Max(A,B)	↑↑

Table S1. Interactive q-statistic values between pairs of risk factors. "↑" denotes A and B bivariate enhance each other; "↑↑" denotes nonlinear enhancement of A and B. The 13 risk factors (TEC, STR, MI, GDP, PRE, SOI, GRO, ELE, TEM, VEG, GEO, SD and SA) are all included.

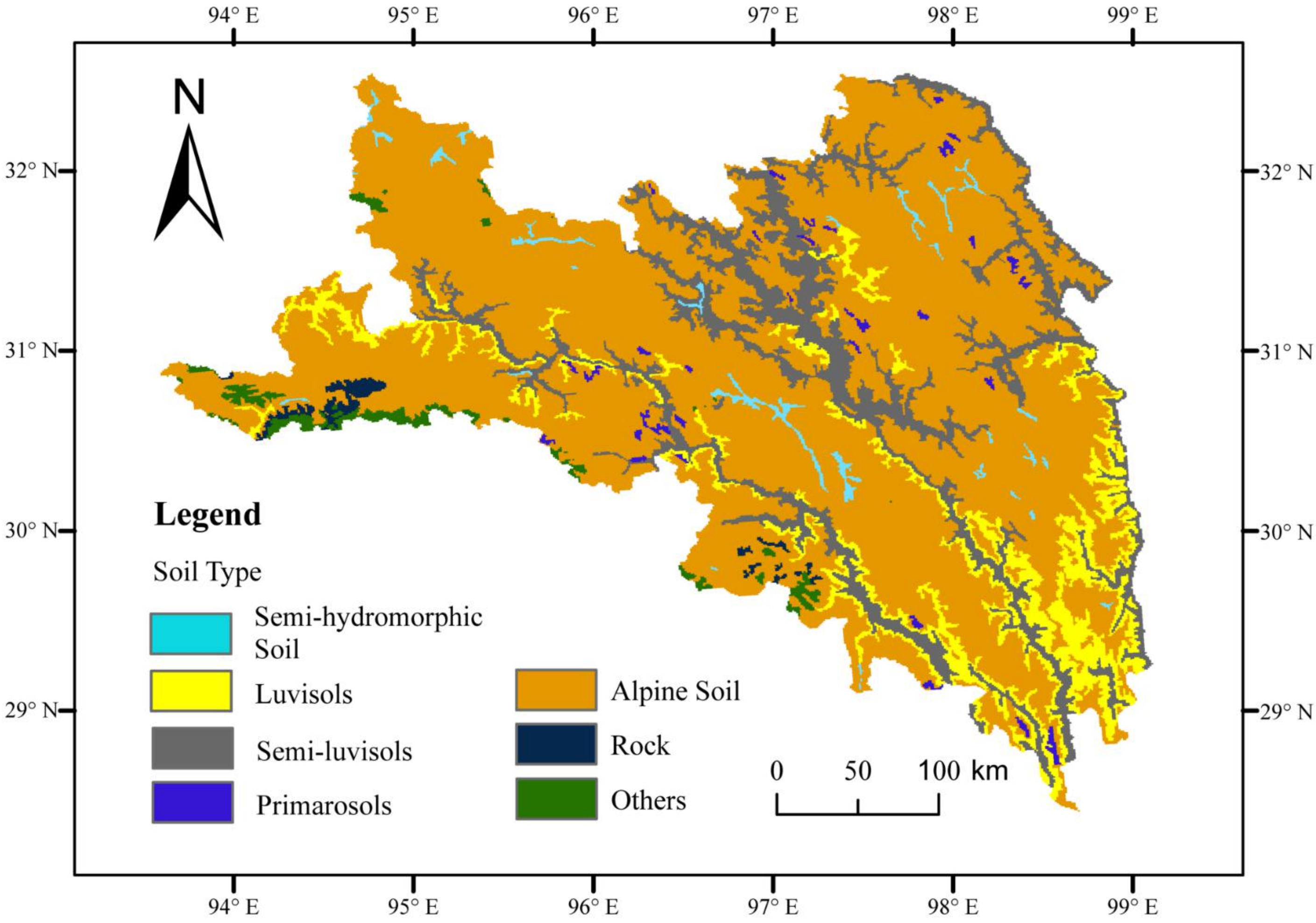

Figure S1. Soil types of the Qamdo prefecture. It was generated by ArcGIS 10.2 (http://www.esri.com/).

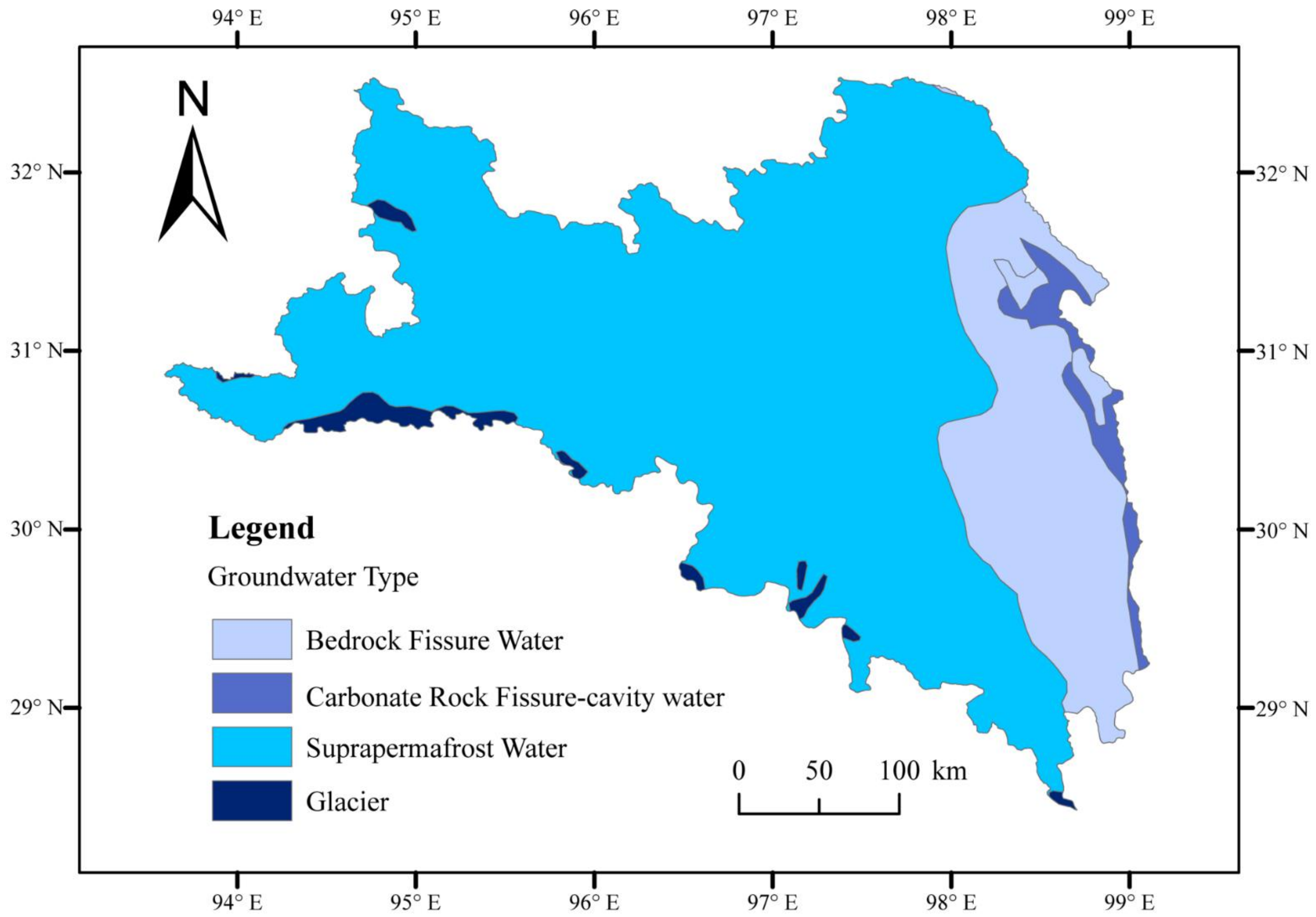

Figure S2. Groundwater types of the Qamdo prefecture. It was generated by ArcGIS 10.2 (http://www.esri.com/).

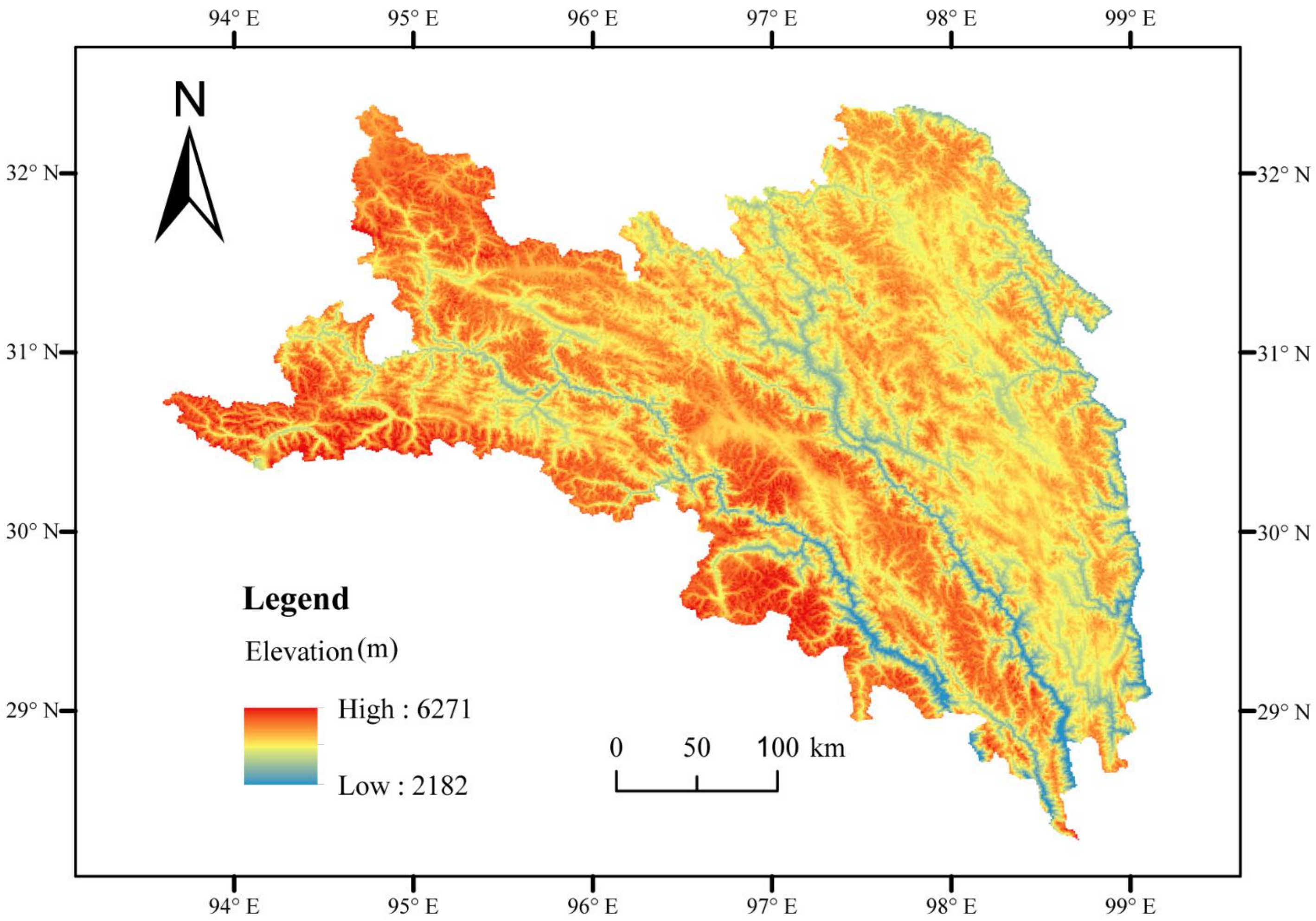

Figure S3. Elevation of the Qamdo prefecture. It was generated by ArcGIS 10.2 (http://www.esri.com/).

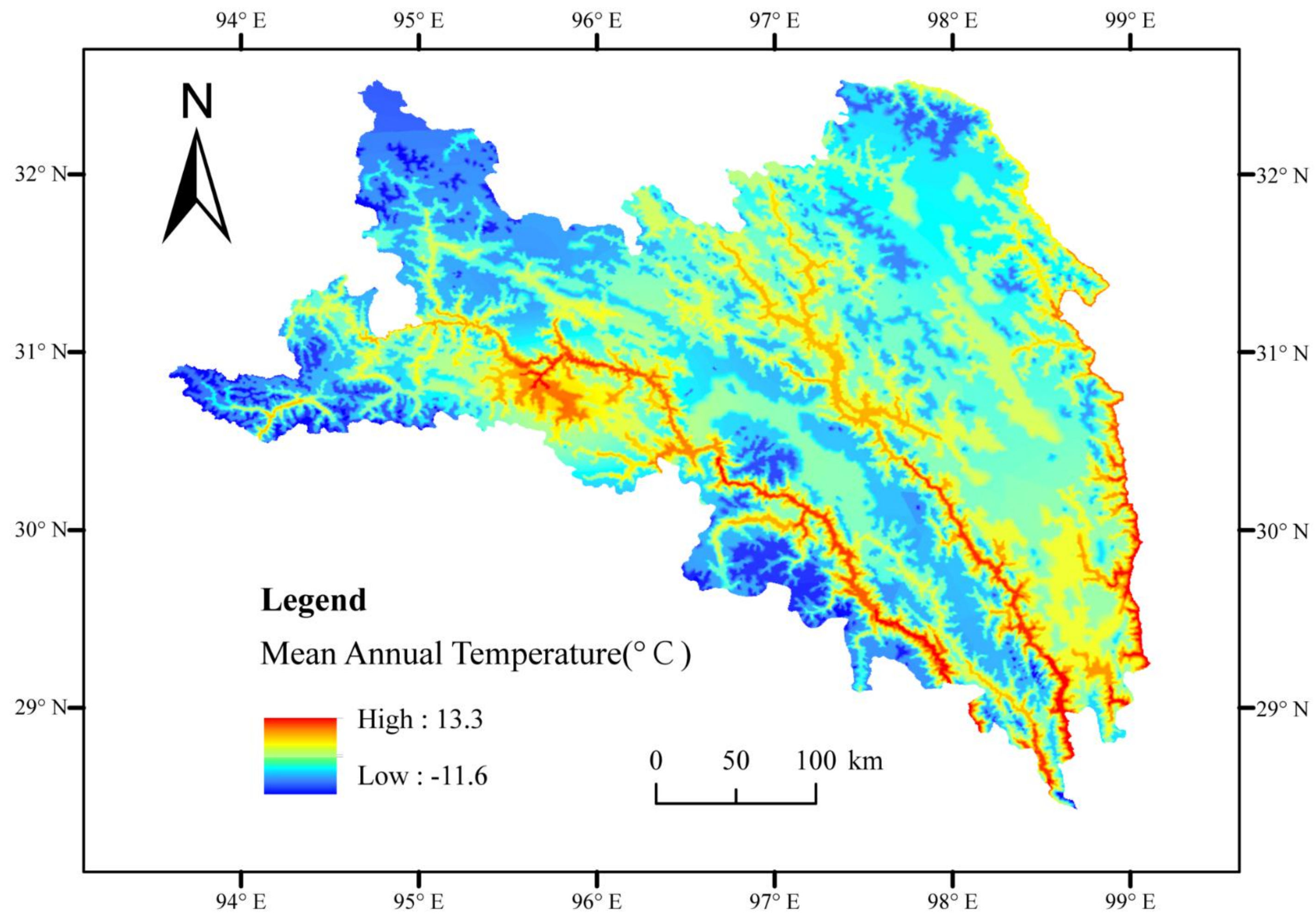

Figure S4. Mean annual temperature of the Qamdo prefecture. It was generated by ArcGIS 10.2 (http://www.esri.com/).

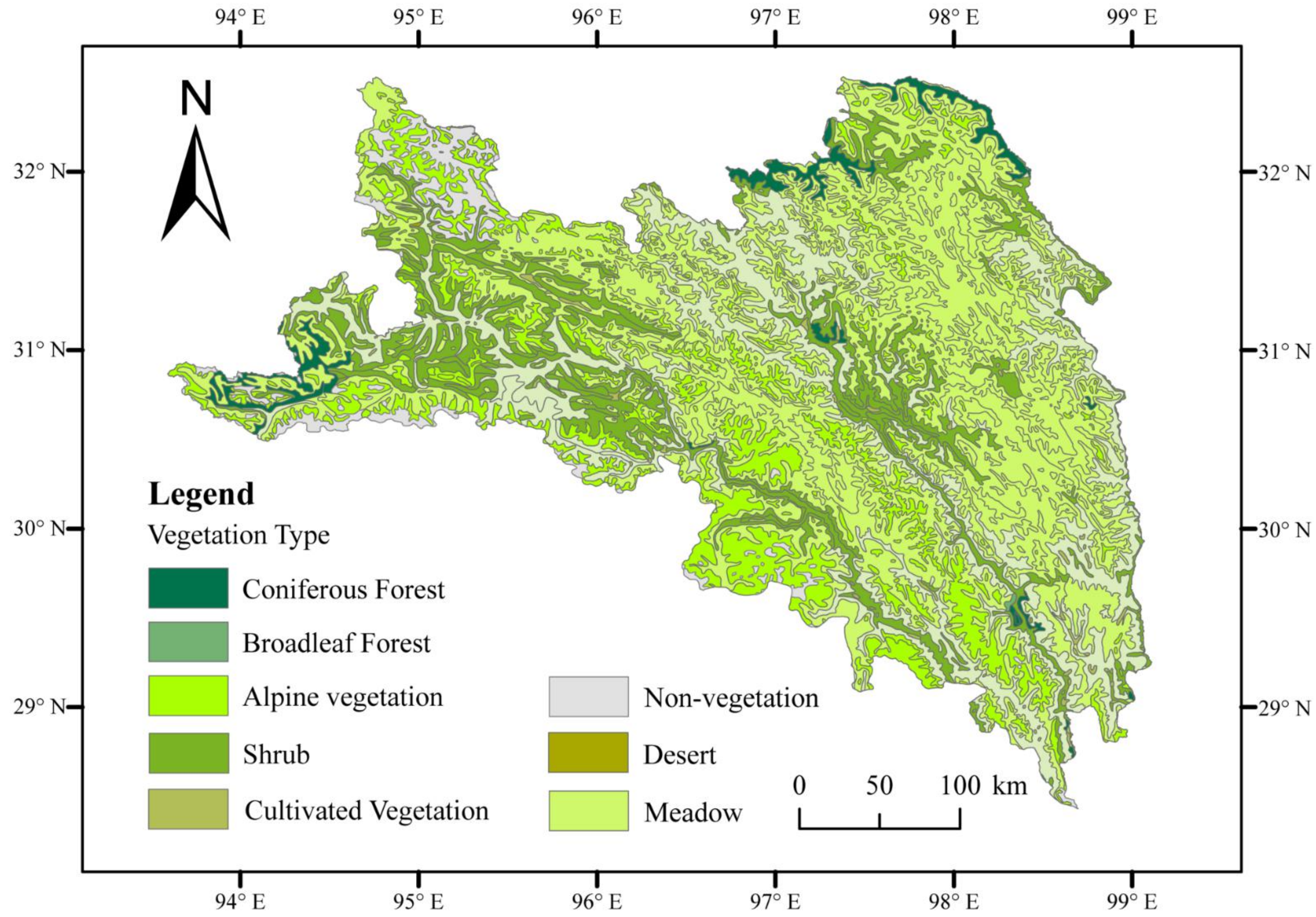

Figure S5. Vegetation types of the Qamdo prefecture. It was generated by ArcGIS 10.2 (http://www.esri.com/).

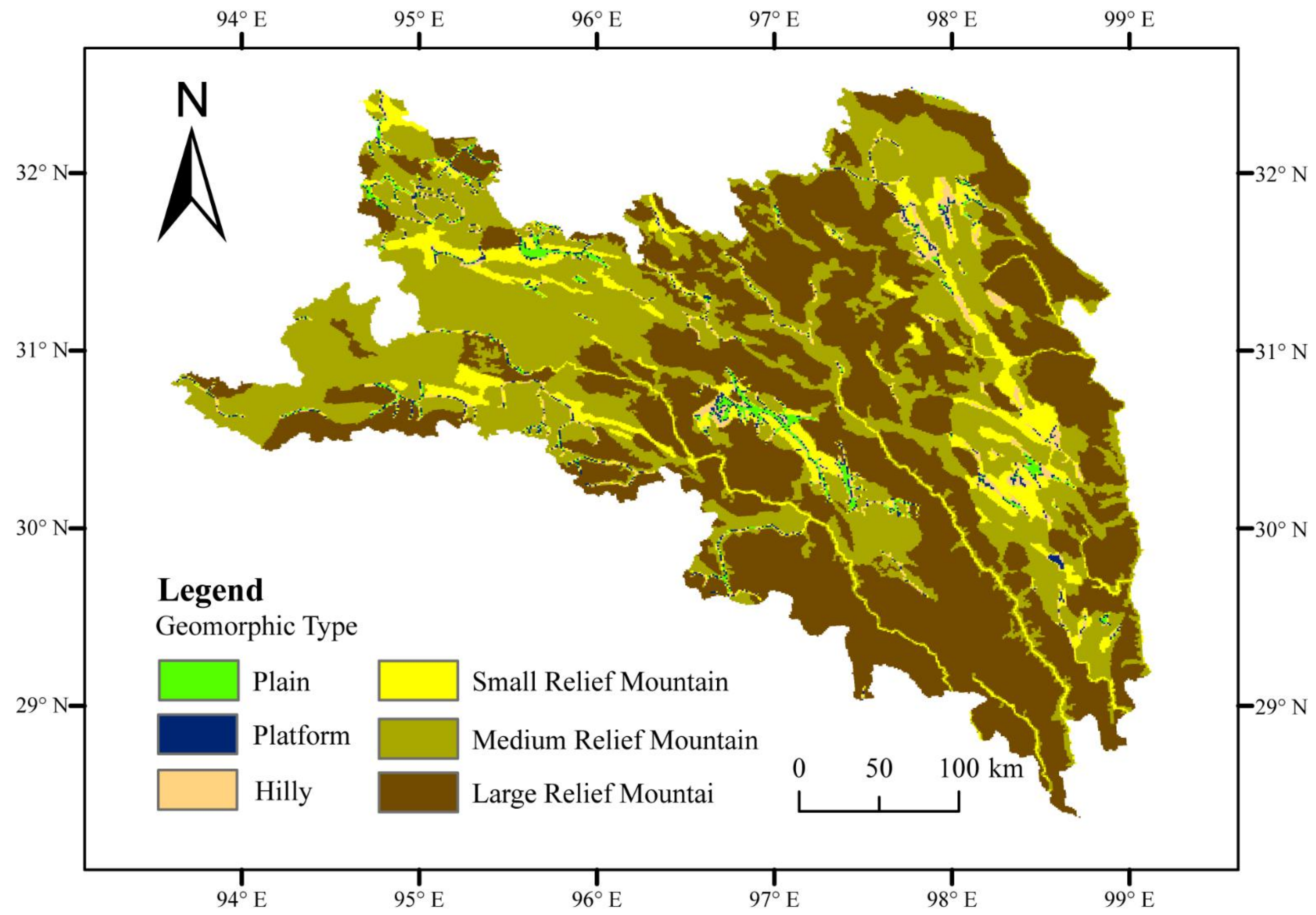

Figure S6. Geomorphic types of the Qamdo prefecture. It was generated by ArcGIS 10.2 (http://www.esri.com/).

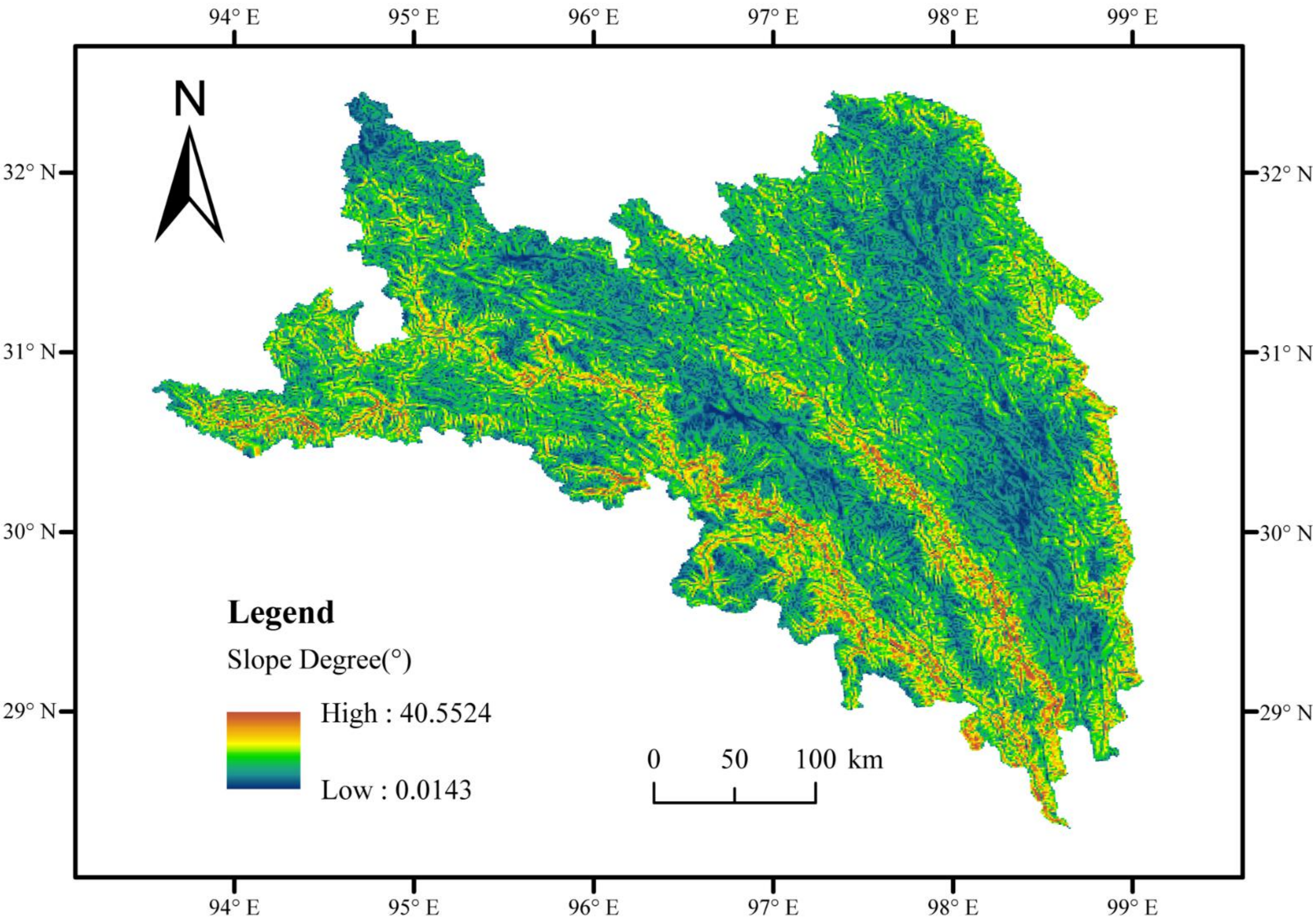

Figure S7. Slope degree of the Qamdo prefecture. It was generated by ArcGIS 10.2 (http://www.esri.com/).

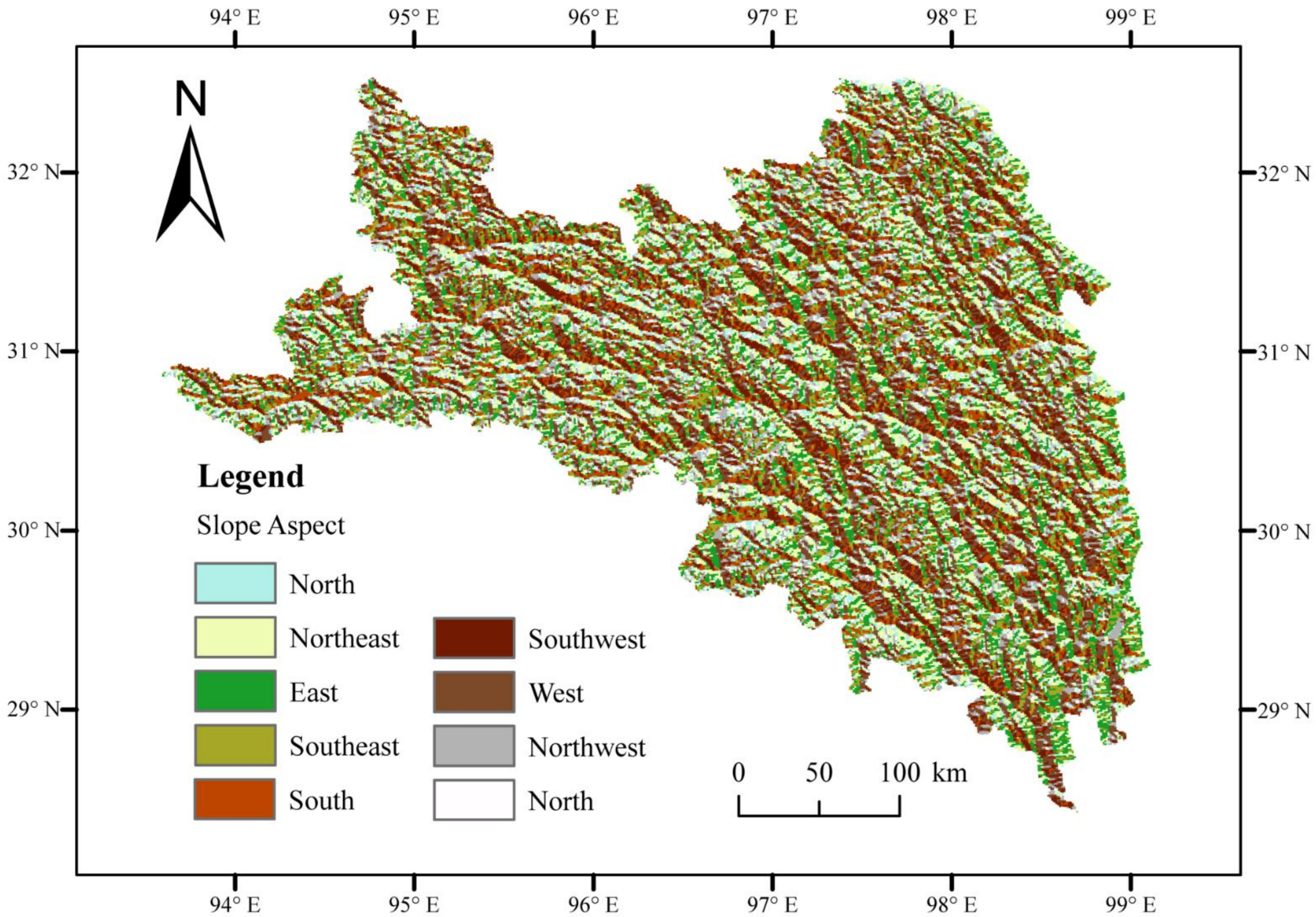

Figure S8. Slope aspect of the Qamdo prefecture. It was generated by ArcGIS 10.2 (http://www.esri.com/).
